# Isolation and characterization of *E. coli* strains causing intramammary infections from dairy animals and wild birds

**DOI:** 10.1080/23144599.2019.1691378

**Published:** 2019-12-03

**Authors:** Karima M Fahim, Elshaimaa Ismael, Hanan S Khalefa, Heba S Farag, Dalia A Hamza

**Affiliations:** a Department of food hygiene and control, Faculty of Veterinary Medicine, Cairo University, Cairo, Egypt; b Department of Veterinary Hygiene and Management, Faculty of Veterinary Medicine, Cairo University, Giza, Egypt; c Department of medicine and infectious disease, Faculty of Veterinary Medicine, Cairo University, Giza, Egypt; d Department of Zoonoses, Faculty of Veterinary Medicine, Cairo University, Cairo, Egypt

**Keywords:** Sub-clinical mastitis, *E. coli*, buffalo, cow, wild birds, antibiotic-resistance

## Abstract

The study was conducted to estimate the prevalence of *Escherichia coli* (*E. coli*) in sub-clinically mastitic (SCM) animals, and in wild and migratory birds which may act as reservoir disseminating such pathogen. Farm hygiene, management and milking procedures were listed through a questionnaire. Thirty lactating cows and 15 lactating buffaloes from five small-scale dairy farms were randomly selected and screened for subclinical mastitis (SCM) using California Mastitis Test (CMT) and somatic cell count (SCC). In addition, 80 teat skin swabs, 5 drinking water samples and 38 wild and migratory bird faecal matter were also collected. All samples were processed for *E. coli* isolation by culturing on Levine’s Eosin Methylene Blue (L-EMB) agar, followed by purification and biochemical identification. Positive samples were subjected to molecular identification and serotyping. In addition, the presence of extended-spectrum beta-lactamase (ESBL) and carbapenemase-producing *E. coli* have been reported by antimicrobial sensitivity testing. *Escherichia coli* were isolated from 7.7%, 50% and 50% of the positive CMT cows’ quarters, cows’ composite and buffaloes’ composite milk samples, respectively. In addition, 14% of cows’ teats, 20% of water samples, 70% of faecal matter from wild bird, and 33.3% of faecal matter from migratory waterfowls were carrying *E. coli*. Serotyping, antibiotic-resistant pattern and phylogenetic analysis have pointed the bearable implication of milking hygiene and wild birds in disseminating *E. coli* strains causing intramammary infections.

## 1. Introduction

Bovine mastitis continues to be a major infection that affects a high proportion of dairy populations worldwide with a negative economic impact on dairy farmers and the dairy industry [1–3]. Furthermore, it possesses a public health concern [4], besides it is considered the single most prevalent cause for antibacterial use in lactating dairy animals [5]. The types of mastitis pathogens are either contagious or environmental organisms [6]. Coliform bacteria are a common aetiology of bovine mastitis. Until recently, *Escherichia coli* (*E. coli*) is the most predominant species and is responsible for more than 80% of coliform mastitis cases [3,7,8] and categorized as a Gram-negative opportunistic environmental bacterium. Mastitis caused by *E. coli* is usually sporadic with clinical signs range from mild to very severe or even fatal forms [9]. Properties and virulence factors specific to *E. coli* strains causing mastitis are still ill-defined, although a high prevalence of phylogroup A was observed in mastitis caused by *E. coli* [10–13]. *E. coli* is mainly a commensal bacterium in the intestinal tract of animals, birds and humans [14]. Although the establishment of biosecurity measures to prevent the entry of infectious agents into dairy farms, dairy herd health still faces several serious threats, regarding open space, which allows uncontrolled access of wild birds into yards, facilities and even feed storage areas [15]. However, few studies focused on wild birds rooming nearby dairy farms [16]. Migratory and non-migratory wild birds serve as reservoirs of coliform bacteria, like *E. coli*, inclosing antimicrobial-resistance genes [17,18]. Water and food contact were suggested to be the primary routes of transmission of resistant bacteria between human or animal and wild birds [19]. Antibiotics are very remarkable for the treatment of bacterial infections in animals and humans [20]. The FDA reported that resistance in *E. coli* is steadily the highest for antimicrobial agents used in human and veterinary medicine [21]. Extended-spectrum beta-lactamase (ESBL) producing *E. coli* is mostly insensitive to lots of commonly used antibiotics causing an increase in the use of last-resort antimicrobial drugs (i.e. carbapenems) during treatment.

The aim of this study was to understand the possible role of wild and migratory birds in carrying and spreading mastitis-causing *E. coli* strains to the dairy farm environment which could represent potential veterinary and public health hazards through infecting the animals and contaminating the milk with antimicrobial-resistant avian strains, in a coincidence of poor milking hygiene.

## 2. Materials and methods

### 2.1. Collection of samples

#### 2.1.1. Milk samples

Milk samples were collected from five dairy farms located in Cairo, Giza, and El-Ismailia governorates in Egypt during the period from August 2017 to January 2018. A total of 15 composite milk samples from 15 lactating cows and 15 composite milk samples from 15 lactating buffaloes were collected. In addition, 60 quarter milk samples from other 15 lactating cows were collected. All animals were apparently healthy.

Milk samples were collected into sterile vials after washing, drying and swabbing of teat ends with 70% ethyl alcohol, and discarding of the first 3–4 streams of milk. Milk samples were subjected to California Mastitis Test (CMT) according to the American Public Health Association (APHA) [22] and somatic cell counting according to Zecconi et al. [23].

#### 2.1.2. Teat swabs

A total of 80 teat skin swabs were collected from 25 cows and 15 buffaloes (40 Hind and 40 fore teat swabs) by a cotton-tipped stick through rotating the cotton-tip on the teat barrel, as described by Piccinini et al. [24].

#### 2.1.3. Drinking water samples

Five water samples (50 ml from each farm) were collected from the drinking troughs of the lactating animals into disposable sterilized test tubes. All samples were stored in an ice box until transported to the laboratory.

#### 2.1.4. Wild and migratory birds faecal matter

A total number of 38 faecal samples were collected from 20 resident wild birds (12 laughing doves “*Spilopelia senegalensis” and* 8 hooded crows “*Corvus cornix*”) and 18 migratory waterfowls (10 eurasian coots “*Fulica atra*” and eight northern shovelers “*Anas clypeata*”) in Giza, Cairo, El-Ismailia and Port-said governorates in Egypt.

Port-Said is one of the Canal Zone governorates and considered a main stop for migratory waterfowls seeking food and rest during the long migration in the spring and fall seasons.

Bird traps were used to capture wild birds around dairy farms and migratory waterfowls during winter migration. Once trapped, faecal swabs were taken, and the birds were released. The swabs were then placed in 2 ml sterile saline (0.9% NaCl) and stored in ice box until transported to the laboratory.

Protocols for the collection of samples were conducted in accordance with the applicable legislation of the Institutional Animal Care and Use Committee, of the Faculty of Veterinary Medicine, Cairo University, Egypt (VetCU10102019087).

### 2.2. Isolation and identification of coliform and E. coli

Loopfuls from positive CMT milk samples were enriched into nutrient broth and incubated aerobically at 37°C for 24 h. The enriched milk samples, teat swabs and loopfuls from water samples were streaked on Violet Red Bile agar (VRB) for isolation of coliforms. Additionally, these samples beside the faecal samples from wild and migratory birds were plated on Levine’s Eosin Methylene Blue plates (L-EMB) for isolation of *E. coli* and incubated at 37ºC for 24–48 h. Suspected colonies of *E. coli* which appeared as dark centred, flat colonies with metallic green lustre were picked up and cultured on agar slants and incubated at 35°C for 18 h and subjected to further identification. Biochemical identification of *E. coli* was carried out according to BAM and Quinn et al. [25,26].

A total of eight representative *E. coli* isolates from buffalo’s milk, cow’s milk, teat skin swabs, drinking water, Laughing dove, Hooded crow, Eurasian coot, Northern shoveler faecal swabs that were isolated from the same locality were selected and serologically identified by slide agglutination test for O-antigen group screening using *E. coli* antisera (Denka Seiken Co., LTD. Chuo-Ku,Tokyo, Japan) according to Kok et al. [27].

### 2.3. Molecular identification of E. coli

All *E. coli* isolates were extracted using DNA Mini Kit (Qiagen, Hombrechtikon, Switzerland) according to the manufacturer’s protocol. For molecular identification of *E. coli*, Primers for *16S rRNA* gene of *E. coli* were selected according to Wang *et al*. [28]. The reaction was carried out using a total volume of 25 μl contain 3 μl of DNA as a template, 5 pmol of each primer and 5μl of 1X PCR master mix (Jena bioscience, GmbH, Germany). The PCR mixtures were then subjected to the following cycling conditions: 50°C (2 min, 1 cycle); 95°C (5min, 1 cycle); 40 cycles of 95°C (45 s), 50°C (1 min), and 72°C (1 min); and 72°C (7 min, 1 cycle) in a thermal cycler (Perkin-Elmer, Waltham, USA).

### 2.4. Antibiotic sensitivity test

The antimicrobial sensitivity for the fore mentioned eight serotyped isolates was evaluated using eight antimicrobials that represent three groups: Carbapenems (*Imipenem*, Ertapenem, Meropenem), Cephalosporins (*Ceftazidime*, C*efotaxime, Ceftriaxone, Cefpodoxime*) and Macrolides (*Azithromycin*).

ESBL production was confirmed by the increase of ≥5 mm in the zone of inhibition for *Cefotaxime/Clavulanic acid* (30/10 mcg) and *Ceftazidime/Clavulanic acid* (30/10 mcg) discs compared to cefotaxime or ceftazidime alone. The interpretation was done according to Clinical and Laboratory Standards Institute (CLSI) [29].

### 2.5. Sequence analysis

The amplified *16S rRNA* fragments of these eight isolates were purified using the QIAquick gel extraction kit (Qiagen, Hombrechtikon, Switzerland) according to the manufacturer’s instructions and sequenced at Promega Lab Technology (Madison, USA) by using the forward and reverse primers listed in table (Table 1). The sequences of the *16S rRNA* gene have been deposited in the GenBank database under the accession numbers mentioned in Table 4. Genes sequenced in this study were compared with the sequences available in public domain using NCBI BLAST server. The *16S rRNA gene* sequences were aligned using CLUSTALW in BioEdit version 7.0.1.4. Phylogenetic analysis was performed with MEGA version 7, using the neighbour-joining approach. The bootstrap consensus tree was estimated from 1000 replicates.

**Table 1.** Primer sequences 16 s *Escherichia coli.*

Primer name (Target gene)	Oligonucleotide sequence (5–3`)*	Amplicon size (bp)
E16S (*16S rRNA*)	F: CCCCCTGGACGAAGACTGAC	401 bp
	R: ACCGCTGGCAACAAAGGATA

**Table 2.** SCC, coliforms and *E. coli* in cows’ and buffaloes’ positive CMT milk samples.

Types of milk samples	Total no.	+ve CMT	Minimum SCC (Cell/ml)	Maximum SCC (Cell/ml)	Mean SCC ±SE (Cell/ml)	Coliforms No. (%)	*E. coli* No. (%)
Quarters – cows	60	26	336 × 10^3^	2 × 10^6^	1.368 × 10^6^ ± 0.085 × 10^6 a^	26 (100%)	2 (7.7%) ^b^
Composite							
Cows	15	14	350 × 10^3^	817 × 10^3^	515 × 10^3^ ± 45 × 10^3 b^	13 (92.9%)	7 (50%) ^a^
Buffaloes	15	4	312 × 10^3^	639 × 10^3^	485 × 10^3^ ± 68 × 10^3^	4 (100%)	2 (50%)

^a,b^ Different superscripts indicate significant difference (*p* < 0.05)

### 2.6. Statistical analysis

Results were analysed using PASW Statistics, Version 18.0 software (SPSS Inc., Chicago, IL, USA). Somatic cell counts were compared by independent sample T-test and expressed as mean ± standard error. Frequencies of isolation were tested with the Chi-square (*x*^2^) (provided that at least 80% of the cells have an expected frequency of 5 or greater, and that no cell has an expected frequency smaller than 1.0), otherwise, the Fisher’s Exact test and the Fisher-Freeman-Halton Exact test (it is the Fisher’s Exact test for contingency tables larger than 2 × 2) were used. A *P*-value <0.05 was considered statistically significant.

## 3. Results

### 3.1. Field observation

Dairy farms included in this study raised from 10 to 100 animals. Animal breeds were Holstein Friesian cows and native buffaloes. Cows age ranged from 6 to 10 years, while buffaloes were around 12 years old. Animals were housed in open yards surrounded with a fence, partially covered with sheds and muddy floors and each yard contains one common water trough for drinking. Each yard is occupied by 10 to 25 lactating cows or buffaloes. All animals were automatically milked twice daily either in-stanchion (10 animals) or in milk parlours (40 to 100 animals). Pre-milking practice was restricted to udder washing and wiping without pre-milking sanitizing teat dip. Only, two farms applied post-milking teat dipping.

### 3.2. California mastitis test (CMT) and milk somatic cell count (SCC)

**Figure 1.**
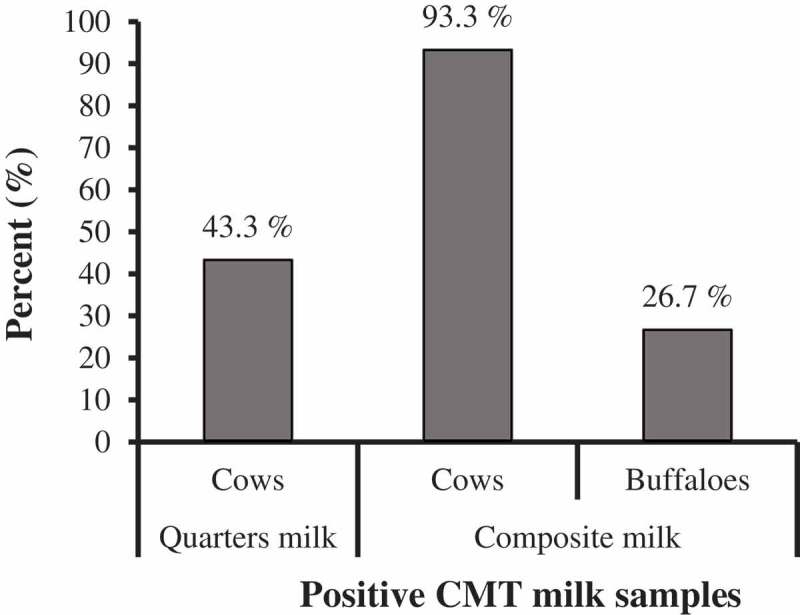
Prevalence of subclinical mastitis in the examined quarter and composite milk samples from cows and buffaloes using California Mastitis Test (CMT).

California mastitis test (CMT) reacted positive with 26 (43.3%) quarters milk samples of cows, 14 (93.3%) composite milk samples of cows, and 4 (26.7%) composite milk samples of buffalos (Figure 1), revealing significant association of subclinical mastitis to cows rather than buffaloes, *x*^2^ (1, N = 30) = 13.889, *p*< 0.001.

**Figure 2.**
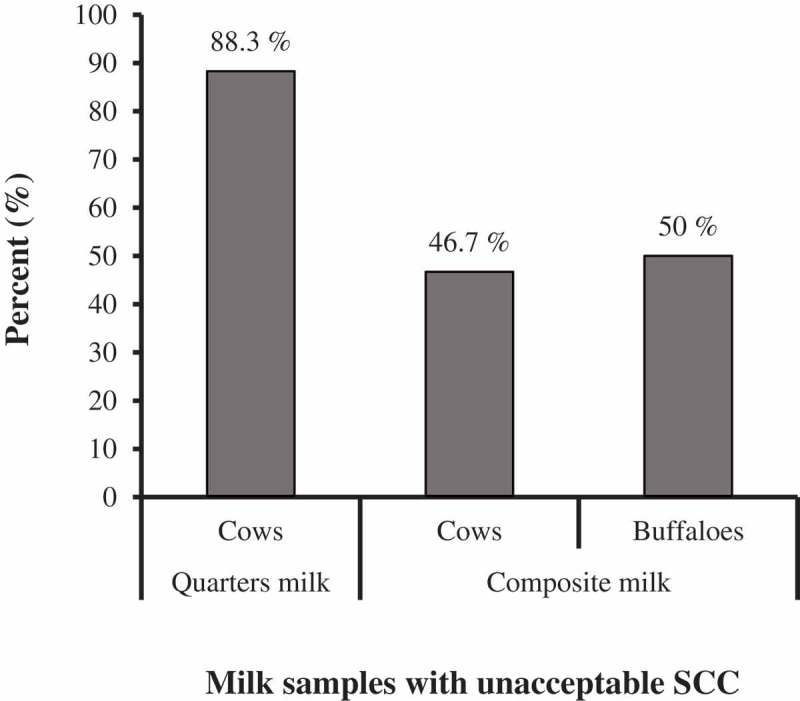
Prevalence of unacceptable milk SCC in examined quarters and composite milk from cows and buffaloes, according to *Egyptian standards of raw milk, 2010.*

Results demonstrated that somatic cell count (SCC) in cows’ quarter milk samples was 8.5 × 10^5^ (±1.9 × 10^5^, ±95% C.I.) cells/ml significantly greater than that in composite milk samples (*t*(69.79) = 8.838, *p*< 0.001) (Table 2). While, somatic cell count (SCC) in composite milk samples of cows was 30 × 10^4^ (±1.9 × 10^5^, ±95% C.I.) cells/ml numerically but not significantly greater than that of buffaloes (*t*(15) = 0.333, *p*= 0.744) (Table 2). According to the Egyptian standards of raw milk [30], subclinical mastitis is considered when SCC exceeds 500 × 103 cells/ml. Consequently, 88.3% of examined cow quarters recorded unacceptable SCC, as well as, 46.7% of cows’ composite and 50% of buffaloes’ composite milk samples (Figure 2). Subclinical mastitis in cows was significantly more likely to be detected in quarter milk samples than in composite ones (*p =*0.002, Fisher’s exact test, FET).

### 3.3. Prevalence of coliforms and E. coli

#### 3.3.1. Milk samples

Bacteriological examination of positive CMT milk resulted in isolation of Coliforms from 26 (100%) quarter milk samples, 13 (92.9%) composite milk of cows, and 4 (100%) composite milk of buffaloes (*p*= 0.409; Fisher-Freeman-Halton Exact test = 2.193). Further examination of positive CMT milk samples resulted in isolation of *E. coli* from 2 (7.7%) quarter milk samples, 7 (50%) composite milk of cows, and 2 (50%) composite milk of buffaloes (Table 2). *Escherichia coli* (*E. coli*) was significantly more likely to be isolated from composite milk samples than from quarter milk samples in cows (*p =*0.004, Fisher’s exact test, FET).

#### 3.3.2. Teat skin swabs and drinking water

Coliforms were isolated from 19 (38%) and 6 (20%) teats of cows and buffaloes, respectively; a difference that was not statistically significant, *x*^2^ (1, N = 80) = 2.828, *p*= 0.093. Further examination resulted in the isolation of *E. coli* from 7 (14%) of cow’s teat skins, but could not be isolated from buffaloes’ teat skins, as listed in Table 3. Coliforms and *E. coli* were isolated from the one-water sample (20%).

#### 3.3.3. Faecal samples of resident wild birds and migratory waterfowls

Bacteriological examination of 38 faecal samples from wild birds and migratory waterfowls resulted in isolation of *E. coli* from 14 (70%) of resident wild birds (laughing doves “*Spilopelia senegalensis*” and hooded crows “*Corvus cornix”*) and 6 (33.3%) of migratory waterfowls (eurasian coot “*Fulica atra*” and northern shoveler “*Anas clypeata”*) (Table 3). Isolation rate of *E. coli* from faeces of wild birds was significantly higher than that from migratory waterfowls, *x*^2^ (1, N = 38) = 5.109, *p*= 0.024.

**Table 3.** Prevalence of coliforms and *E. coli* on teat skin swabs, drinking water samples and birds’ faecal swabs.

Samples	Total no.	Coliforms No. (%)	*E. coli* No. (%)
**Cows:**			
● Fore teats	25	10 (40%)	3 (12%)
● Hind teats	25	9 (36%)	4 (16%)
● **Total**	**50**	**19 (38%) ^a^**	**7 (14%)**
**Buffaloes:**			
● Fore teats	15	4 (26.67%)	0
● Hind teats	15	2 (13.33%)	0
● **Total**	**30**	**6 (20%) ^a^**	**0**
**Drinking water:**	**5**	**1 (20%)**	**1 (20%)**
**Resident wild birds:**			
● Laughing Doves (*Spilopelia senegalensis*)	12		8 (66.6%)
● Hooded Crow (*Corvus cornix*)	8		6 (75%)
● **Total**	**20**		**14 (70%) ^a^**
**Migratory waterfowls:**			
● Eurasian Coot (*Fulica atra*)	10		3 (30%)
● Northern Shoveler (*Anas clypeata*)	8		3 (37.5%)
● **Total**	**18**		**6 (33.3%) ^b^**

^a,b^ Different superscripts indicate significant difference (*p* < 0.05)

**Table 4.** Serotyping of *E. coli* isolates and their antibiogram.

		Dairy farms				Resident birds		Migratory birds	
Samples		Cow milk	Buffalo milk	teat skin	Drinking water	Laughing dove	Hooded crow	Eurasian coot	Northern shoveler
*E. coli* serotypes		O166	O146	O146	O1	O166	O78	O18	O158
Accession number		MK503655	MK503954	MK503972	MK503975	MK503974	MK503973	MK503976	MK503978
Antibiotics (mcg*)									
Carbapenems	Imipenem (10)	S	S	I	S	I	I	I	S
	Ertapenem (10)	S	S	R	R	I	I	I	S
	Meropenem (10)	S	S	R	I	S	S	S	S
Cephalosporins	Ceftazidime (30)	I	I	S	R	R	R	R	I
	(Cephotaxime) (30)	R	R	R	R	R	R	R	R
	Ceftriaxone (30)	R	S	R	R	R	R	R	S
	Cefpodoxime (10)	R	R	R	R	R	R	R	R
Macrolides	Azithromycin (15)	I	I	S	R	R	R	R	I
ESBL producer	Ceftazidime/Clavulanic acid (30/10)	-	-	-	-	-	ESBL	-	-
	Cefotaxime/Clavulanic acid (30/10)	-	ESBL	-	ESBL	ESBL	ESBL	ESBL	-

* **mcg**: Micrograms.

All *E. coli* isolates were molecularly confirmed by PCR using Primers for 16S rRNA gene of *E. coli*.

### 3.4. Serotyping of E. coli

Results in Table 4 revealed different *E. coli* serotypes. *E. coli* O166 serotype was isolated from both cow’s milk and laughing dove’s faecal swab, and O146 serotype was isolated from both buffalo’s milk and teat skin. Serotypes O1, O78, O18 and O158 were isolated from drinking water, faecal swab of hooded crow, faecal swab of eurasian coot and of northern shoveler; respectively.

### 3.5. Antibiotic sensitivity of E. coli isolates

The antibiogram demonstrated that the Carbapenems group of antibiotics showed the greatest efficacy against most of the *E. coli* isolates. Resistance to Carbapenems group appeared from the environmental *E. coli* serotypes isolated from teat skin and drinking water. All the eight *E. coli* isolates displayed resistance against both *Cefotaxime* and *Cefpodoxime* antibiotics.

### 3.6. Sequence analysis

**Figure 3.**
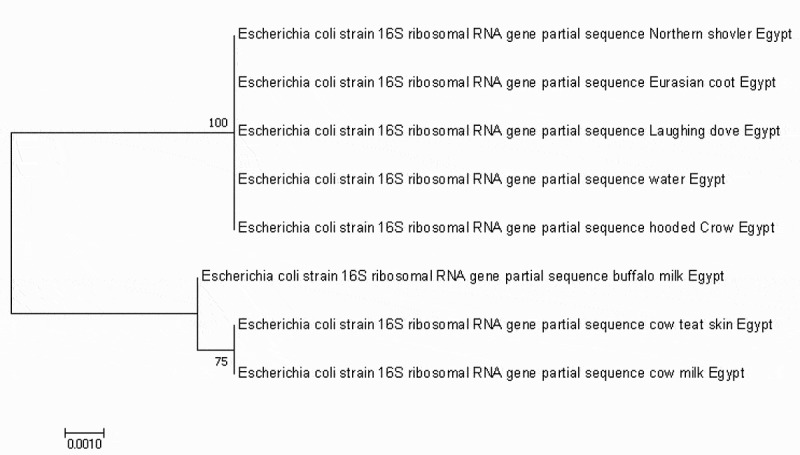
Phylogenetic analysis using neighbour-joining method based on the partial sequence of *16s rRNA*. The bootstrap consensus tree demonstrates the evolutionary history of the obtained *E. coli* strain. The tree was constructed by Mega 7 software.

Phylogenetic analysis of *16S rRNA* gene sequences of these eight *E coli* isolates showed100% homology between isolates from northern shoveler, eurasian coot, laughing dove, hooded crow and water as shown now in the phylogenetic tree (Figure 3).

## 4. Discussion

The Small-scale dairy farming is a significant economic sector for the enhancement of agriculture and livelihoods in developing countries [31,32]. It begins from 2, 15 up to 20 or even 50 milking animals [33–35]. Mastitis is a multi-factorial production disease and possess a threat for small-scale producers as its prevalence exceeded 50% according to FAO, 2014 report [36]. In subclinical mastitis, milk and udder appear normal, but there is decreased milk production, positive reactivity with CMT, high milk somatic cell count, presence of pathogens in the milk, and altered milk composition [37–39]. California Mastitis Test (CMT) is a common screening test used for detection of subclinical mastitis and it revealed that 43.3% of quarter milk samples were affected with subclinical mastitis. These results agreed with that reported by Sori et al. [40] (40.6%) and Ayano et al. [41] (43.02%). A higher prevalence (60%) was reported by Ayano et al. [42], and a lower prevalence ranged from 18.14% to 23.25% were reported by Bangar et al. [43], Patel and Trivedi [44], and Singh et al. [45]. On the other hand, somatic cell count (SCC) is the gold standard for confirming subclinical mastitis [46]. Healthy udder has milk SCC ranges from 50,000 and 100,000 cells/ml, up to 200,000 cells/mL, while in subclinical mastitis the SCC exceeds 200,000 cells/ml [47]. In Egypt, subclinical mastitis is confirmed when SCC exceeds 500,000 cells/ml [30]. In Table 2, the average SCC of quarter milk samples and composite milk samples were similar to the average value (9.24 ± 2.0 × 105 cells/ml) reported by Skrzypek et al. [48] and average (44.8 ± 0.26 x 104 cell/ml) reported by Mohamed et al. [49], respectively.

Coliforms are environmental mastitis-causing pathogens, they are primarily present in moisture, mud, faeces and other organic matter present around the animals. *Escherichia coli* is the most commonly isolated coliform species from intramammary infections and clinical mastitis; besides it has a significant public health importance as it causes diarrhoea in human [40,50,51]. Prevalence of coliform infections (Table 2) disagreed with Hawari and Al-Dabbas [52] who detected coliforms in the milk of 31.9% of the mastitic quarters. On the other side, El-Khodery and Osman [53] detected coliforms in 80.36% of the affected buffaloes, but Sayed [54] recorded lower coliform prevalence (21.78%) in the examined milk samples. *Escherichia coli* was isolated form 7.7% quarter milk samples which is lower than that reported by Verma et al. [55] (21.28%). *Escherichia coli* has been detected in 50% of composite milk of buffaloes and cows which agreed with Mansour et al. [56] who found *E. coli* in 47% of machine-milked samples.

Poor hygiene, husbandry and milking technique could predispose to environmental mastitis as well as milk contamination [57]. Gram-negative bacteria pass into the mammary gland through teat canal [50]. Previous studies stated that regular teat dipping for controlling of mastitis is not a common practice at small-scale farms, which agrees with the findings of our study [58]. *Escherichia coli* was detected on the skin of 14% of cows’ teats as shown in Table 3, which poses a critical threat to the animals as well as consumer health as concluded by Galiero [59]. Teat skin act as the primary reservoir of microbes that can infect milk during milking [60]. Moreover, Jones [61] showed that the incidence of clinical mastitis was related to bacterial populations on teat ends.

As *E. coli* is a ubiquitous organism, it colonizes the gut of birds as well as mammals [62]. Recent studies assumed that wild birds play a crucial role in spreading of foodborne bacteria among dairy farms [63,64]. The practice of feeding dairy animals in open yards and accumulation of their manure lead to the attraction of wild birds into dairy farms [65]. Results in Table 3 showed that faecal matter of 70% of wild resident birds and 33.3% of migratory waterfowls contained *E. coli*. Similar studies isolated identical *E. coli* strains from excreta of wild birds collected from two dairy farms with 32.5 km distance apart, on the same sampling time [64,66].

Serotyping of *E. coli* isolates from different sources in the same locality revealed that O-146 and O-166 serotypes were the most frequent strains (Table 4). The O-146 type was isolated from both buffalo milk and teat skin and this indicated that contamination of teat skin with environmental pathogens possess a risk for occurrence of mastitis [61]. The same serotype was isolated by Wenz [12] from milk samples of cows with acute coliform mastitis and by Wang et al. [67] from ducks in China. *Escherichia coli* O-166 serotype was isolated from both cow milk and dove excreta which shows the probable role of wild birds in acting as reservoir and spreader of mastitis-causing pathogens among dairy farms [65–67]. Furthermore, the same serotype was previously reported to cause an outbreak of gastroenteritis in humans in Japan [68], which displays the significance of this serotype on the public health. *Escherichia coli* serotypes O-1, O-18, O-78 and O-158 which were isolated from drinking water, migratory ducks (coot), wild birds (crows) and migratory shoveler duck, respectively, were known as the most frequent Avian Pathogenic and Avian Faecal *E. coli* (APEC and AFEC) serotypes infecting chickens, turkeys, ducks or other birds [67,69–71]. Moreover, O-18, O-78 and O-158 were previously isolated by Wenz et al. [12] from milk samples of cows suffering acute coliform mastitis with varying systemic disease severity.

The antibiotic resistance of *E. coli* is of a particular concern because it is the most common Gram-negative pathogen in humans [72]. In addition, resistant *E. coli* strains could transfer and acquire antibiotic-resistance elements to and from other bacteria [73]. In the current study, the serotyped *E. coli* isolates showed resistance against most of Cephalosporins group of antibiotics (Table 4). On contrary, all *E. coli* isolates showed considerable sensitivity to Carbapenems group of antibiotics except that isolated from teat skin and water (Table 4). Carbapenems, like imipenem or meropenem, are considered the “last line of defense” in the treatment of infections caused by multi-resistant Gram-negative bacteria. Previous studies stated that bacteria isolated from wild and migratory bird populations acquired resistance although they are not directly affected by antibiotic practices [74]. The number of studies describing the prevalence of ESBL-producing *Enterobacteriaceae* has increased rapidly around the world [75]. Out of the eight *E. coli* isolates, five ESBL producing *E. coli* isolates were detected (two from wild resident birds, one from migratory waterfowls, one from buffalo milk, and one from drinking water). In recent years, ESBL-producing *Enterobacteriaceae* have been recovered from different sources in the community, including cattle, chickens, and raw milk [76–78]. Interestingly, ESBL-producing *E. coli* in wildlife was firstly reported shortly after their isolation from livestock farming which could suggest the spread of ESBL-*E. coli* into the environment through manure [79], and subsequently spread to different ecological niches [80].

These results were also confirmed by sequence analysis of *16S rRNA* gene of these eight isolates. The phylogenetic analysis revealed that the sequence from northern shoveler, eurasian coot, laughing dove, hooded crow and water were identical to each other suggesting the possible role of migratory birds to contaminate water sources in the farm since they were found in the same clade.

## 5. Conclusion

The study showed that faecal matter of wild and migratory birds could be considered as a potential risk factor for disseminating pathogenic and/or resistant *E. coli* in a dairy farm environment, leading to contaminating teat skin of lactating animals, triggering mastitis and/or milk contamination, and threatening animal and human health. Frequent removal of manure could lessen wild bird density inside dairy farms. Milking hygiene practices, as; washing worker hands, rinsing udder and teats in sanitizing solution, then drying them, followed by effective germicidal teat dip could reduce pathogenic bacterial load on the udder skin and prevent mastitis-causing pathogens from populating the environment or teat skin of the animals. Further research should be carried out to explore other environmental risk factors responsible for intramammary infections within dairy herds.
